# Glycine attenuates sepsis-induced white matter injury by modulating gut microbiota

**DOI:** 10.3389/fmolb.2025.1733207

**Published:** 2026-01-08

**Authors:** Jingfei Liu, Li Zhang, Chunyang Feng, Ye Li, Huiling Wu, Xueer Wang, Dong Li

**Affiliations:** 1 Department of Neonatology, The First Affiliated Hospital of Dalian Medical University, Dalian, China; 2 Department of Neonatology, The Second Affiliated Hospital of Dalian Medical University, Dalian, China

**Keywords:** sepsis, white matter injury, glycine, gut microbiota, C5aR1

## Abstract

Sepsis poses a significant threat to preterm infants and is a leading cause of white matter injury (WMI); however, effective therapeutic strategies remain limited. Recent studies suggest that gut microbiota dysbiosis contributes to sepsis-induced systemic inflammation and neurological damage. After treating mice with LPS-induced sepsis with glycine, we evaluated pathological changes in the brain and ileum by HE staining and analyzed gut microbiota composition by 16S rRNA gene sequencing. Inflammatory cytokine levels in brain and ileal tissues were quantified by ELISA. Transcriptomic profiling was performed to identify differentially expressed genes and enriched pathways in the brains of septic mice with WMI. Additionally, protein expression levels of occludin, Iba-1, BMP, and C5aR1 were assessed by IHC and Western blotting. The study demonstrates that sepsis induces WMI. Glycine alleviated intestinal dysbiosis, restored the expression and function of intestinal tight junction proteins, and reduced pro-inflammatory cytokine levels in both ileal and brain tissues. Moreover, glycine attenuated microglial activation, as evidenced by decreased Iba-1 expression, and preserved myelin integrity by preventing the loss of MBP in the brain. Transcriptomic analysis revealed significant upregulation of C5aR1 in brain tissue associated with sepsis-induced WMI. Collectively, these findings indicate that glycine represents a promising therapeutic strategy for the prevention and treatment of sepsis-associated WMI, and that targeting the C5aR1-mediated complement pathway may offer a novel approach to mitigate neuroinflammation and white matter damage.

## Graphical Abstract

**Figure:**
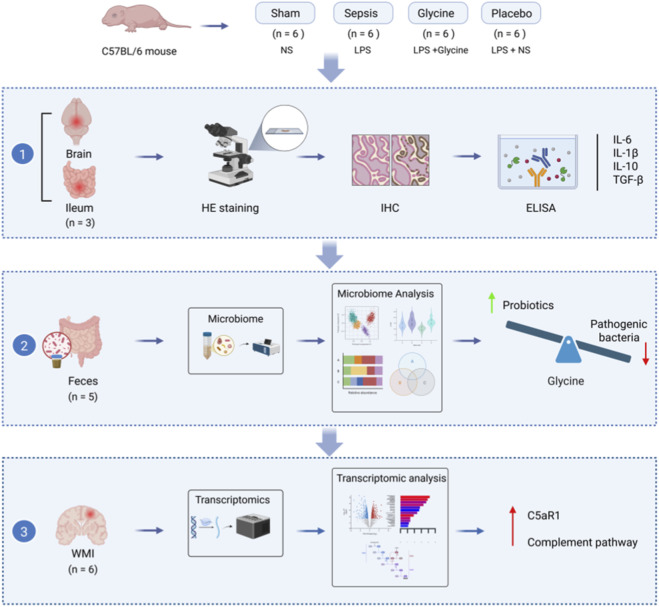

## 1 Introduction

Preterm infants are particularly vulnerable to sepsis, a systemic inflammatory response syndrome that increases morbidity and mortality (Celik et al., 2022; Flannery et al., 2025). Sepsis is responsible for 13% of all neonatal deaths worldwide and is the third leading cause of neonatal mortality (Korang et al., 2021). WMI is the one of the common complicates of sepsis (Mazeraud et al., 2018). About 50%–80% of the long-term consequences of premature newborns with WMI are related to learning deficits, visual and auditory impairments, and other neurodevelopmental abnormalities (Andersen et al., 2025; Taneri et al., 2025; Locke and Kanekar, 2022). The current treatment methods for WMI in premature infants are limited, necessitating an urgent search for novel approaches to therapy in clinical and scientific research.

Sepsis is frequently accompanied by gastrointestinal dysfunction, which heightens mortality risk (Zuo et al., 2023). Intestinal microbiota dysbiosis is common in septic patients and may contribute to disease onset or progression (Mao et al., 2023). Microbiota plays a pivotal role in host-microbe interactions (Kamble et al., 2024), and are linked to various metabolic and inflammatory disorders, including sepsis (Yang et al., 2024; Kamble et al., 2025). Emerging evidence underscores that modulating gut microbiota composition can attenuate systemic inflammation, highlighting its therapeutic potential in sepsis (Liu L. et al., 2024). Restoring intestinal microbial balance represents a promising strategy for preventing and treating sepsis-induced WMI. Microbial metabolites, such as glycine, synthesized by gut bacteria including *Lactobacillus*, *Bifidobacterium*, and *Clostridium* (Iwamoto et al., 2021). As a non-essential amino acid, glycine is crucial for detoxification, antioxidation, and other vital physiological processes (Aguayo-Cerón et al., 2023; Zhang et al., 2025). Reduced glycine levels have been linked to intestinal dysfunction in preterm models, as demonstrated in studies showing decreased urinary glycine in preterm pigs (Alinaghi et al., 2020). Glycine acts as an inhibitory neurotransmitter that modulates both excitatory and inhibitory synapses (Bi et al., 2022; Holeček, 2025) and may influence inflammatory signaling along the gut-brain axis via systemic humoral and cellular immune pathways (Agirman et al., 2021). The gut-brain axis involves complex crosstalk among the gut microbiota, intestinal barrier, immune system, vagus nerve, enteric nervous system, and central nervous system (Loh et al., 2024), and growing data implicate microbiota dysbiosis in the pathogenesis of neurodevelopmental and neurodegenerative disorders (Liu et al., 2022).

Our preliminary investigations revealed that preterm infants diagnosed with late-onset sepsis exhibited significantly lower fecal glycine concentrations compared to healthy controls (Liu J. et al., 2024). Follow-up assessments further indicated that the majority of these infants developed WMI. Recent studies have shown that colitis-associated gut microbiota can modulate brain glycine levels through microbial metabolic pathways, influencing anxiety-like behaviors, sensorimotor gating, and social function (Morozova et al., 2022). Glycine serves not only as a classical inhibitory neurotransmitter but also as a critical mediator linking gut microbial metabolites to central nervous system activity (Cai et al., 2025). Acting as a potential metabolic signaling molecule in the gut microbiota-brain axis, glycine may serve as both a biomarker and a driver of disrupted neurodevelopment when its levels are dysregulated (Tosi et al., 2023). Considering that glycine is produced by select gut microbes and contributes to the regulation of host immune homeostasis (Ji et al., 2022), its role in linking sepsis-induced dysbiosis to neuroinflammatory processes warrants further investigation. Given its important role in immune response regulation and potential influence on gut microbiota and composition, we hypothesized that glycine acts as a mediator in gut-brain communication during sepsis pathogenesis. We aimed to identify the mechanisms behind glycine’s benefits, with a focus on its ability to relieve sepsis-induced WMI.

## 2 Experimental procedures

### 2.1 Animal models

The Animal Ethics Committee of the Animal Experiment Centre at Dalian Medical University (Approval No. AEE24088) granted approval for this research. C57BL/6 mice were purchased from Liaoning Changsheng Biotechnology Co., Ltd. (Number of quality certification: 210726241102709171) and subsequently reared in the SPF-grade breeding facility of the SPF Animal Experiment Centre at Dalian Medical University. The mother mice were allowed to nurse their offspring, access water and consume food freely. A 12-h light/dark cycle was established to simulate the diurnal pattern, with the ambient temperature maintained at 23 °C ± 1 °C and the relative humidity sustained at 45% ± 5%.

**FIGURE 1:**
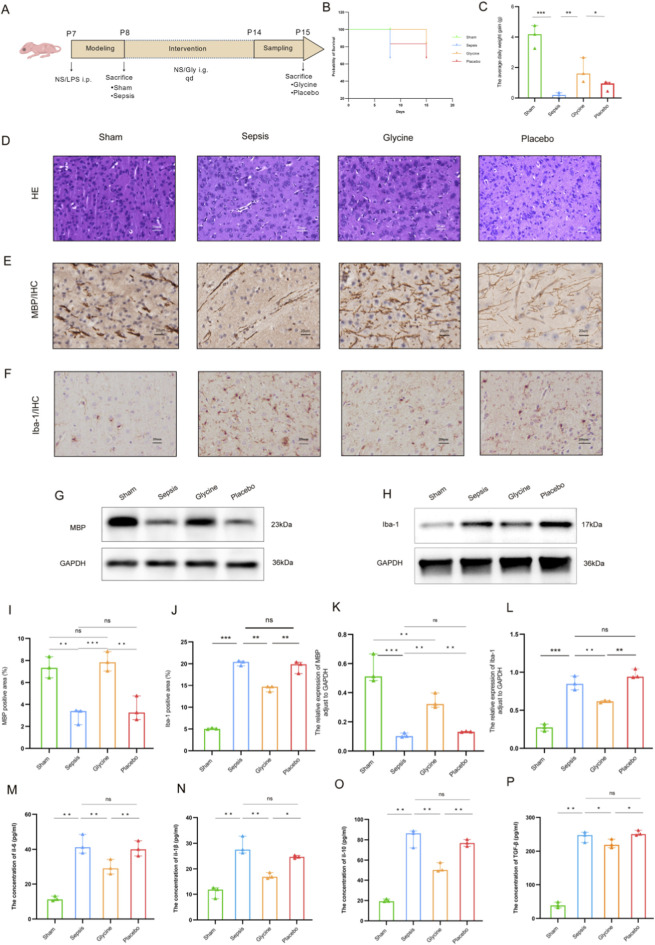
Glycine alleviates the WMI and inhibits microglial activation in septic mice. **(A)** Experimental timeline of sepsis induction and glycine Intervention in neonatal mice. **(B)** Survival curve of neonatal mice (n = 6 per group). **(C)** The daily body weight gain in mice (n = 3 per group). **(D)** HE staining of the brain’s white matter area in mice from each group. **(E)** MBP expression in the brain white matter region of mice in each group was identified by IHC. **(F)** IHC analysis revealed positive expression of Iba-1 in the brain white matter region of mice in each group. **(G,H)** Western blot analysis of MBP **(G)** and Iba-1 **(H)** protein levels in ileal tissue. **(I,J)** Quantification of MBP-positive **(I)** and Iba-1-positive **(J)** area fractions in cerebral white matter by IHC. **(K,L)** Densitometric analysis of MBP **(K)** and Iba-1 **(L)** protein expression in brain tissue by Western blot, normalized to GAPDH. The concentrations of inflammatory cytokines **(M)** IL-6, **(N)** IL-1β, **(O)** IL-10, and **(P)** TGF-β in brain tissues were quantified by ELISA kits. Data are presented as mean ± SD. For HE staining and IHC staining: magnification: 400×; scale bar: 20 μm *P < 0.05; **P < 0.01; ***P < 0.001; ns for no statistical significance. Data form **(D–P)** are representative of three independent experiments.

Seven-day-old (P7) male mouse pups (weight: 3.2–5.5 g) were randomly divided into four groups (n = 6 in each group): Sham group, Sepsis group, Glycine group, and Placebo group. Another group of healthy P15 mice (sham2 group, n = 6) was included in the study as a baseline reference for the analysis of the gut microbiota. There was no significant difference in the initial body weight among the groups (P > 0.05). According to the previous method, an LPS-induced sepsis model was established (Chen et al., 2022; Mao et al., 2023). Mice were intraperitoneally injected with lipopolysaccharide (LPS, 10 mg/kg) once, while the sham operation group was given the same amount of normal saline (NS). Subsequently, for seven consecutive days, the glycine group was treated qd with a glycine solution (0.125 mg/g) via oral gavage, whereas the placebo group received an equivalent volume of NS (Rom et al., 2020) (Figure 1A). Following administration, the pups were returned to their mother for nursing. Their survival status and body weight gains were monitored daily (Figures 1B,C).

### 2.2 Sample collection

Mice were deeply anesthetized by inhaling isoflurane (3%–5%) in 100% oxygen, followed by cardiac perfusion with 4% paraformaldehyde (PFA) or normal saline solution. After hematoxylin-eosin (H&E) staining and immunohistochemistry (IHC), the tissue sections were preserved in 4% PFA for histological and molecular studies. Samples were stored at −80 °C for Enzyme-Linked Immunosorbent Assay (ELISA), Transcriptomics analysis and Western blotting. Intestinal fecal samples were aseptically collected, quickly transferred into sterile EP tubes, snap-frozen in liquid nitrogen, and preserved at −80 °C within 1 h of collection for further analyses.

In this study, fecal samples were collected at P8 and P15. P8 was selected to capture early alterations in the gut microbiota during the acute phase of sepsis. Glycine was administered daily from P8 to P14, and P15 was chosen to assess the cumulative effects of treatment after its completion. By P15, the gut microbiota typically reaches higher biomass and a more stable community structure, which yields higher quality and more reproducible 16S rRNA sequencing data.

### 2.3 H&E and IHC staining

After fixation in 4% PFA, brain and ileal tissues were embedded in paraffin. Sections (4 μm thick) were cut and stained for histological analysis. Pathological changes in ileal tissue sections were examined under light microscopy. Histopathological evaluation was performed using a standardized histological scoring system. Five distinct parameters were assessed: (1) severity of epithelial injury; (2) depth of ulceration; (3) degree of submucosal edema; (4) extent and depth of lymphomonocytic infiltration; (5) intensity and pattern of neutrophilic and eosinophilic infiltration (Tang et al., 2019). Ileal tissue sections were immunostained with primary antibodies against occludin (1:1000, Abcam, United Kingdom), C5aR1 (1:1000, Abcam, United Kingdom). Brain sections were stained with antibodies against MBP (1:2000, Abcam, United Kingdom), Iba-1 (1:1000, Abcam, United Kingdom), and C5aR1 (1:1000, Abcam, United Kingdom). For each mouse, three white matter regions were randomly selected, and the percentage of immunopositive area was quantified using Fiji (version 2.3.0; https://fiji.sc/).

### 2.4 16S rDNA sequencing

Due to animal mortality, fecal samples from five mice per group were used to ensure equal sample sizes across all groups. Total fecal microbial DNA was obtained through the Fecal Genome DNA Extraction Kit (AU46111-96, BioTeke, China) according to the manufacturer’s instruction manual. The DNA was quantified by Qubit (Invitrogen, United States). Total DNA was amplified by PCR using the universal primer 341F/805R (341F: 5′-CCTACGGGNGGCWGCAG-3’; 805R: 5′-GACTACHVGGGTATCTAATCC-3′). The PCR amplification conditions were pre-denaturation at 98 °C for 30 s, denaturation at 98 °C for 10 s, annealing at 54 °C for 30 s, extension at 72 °C for 45 s and 32 cycles. The final extension was at 72 °C for 10 min. The PCR product was purified using AMPure XP Beads (Beckman Coulter Genomics, Danvers, MA, United States) and quantified using Qubit (Invitrogen, United States). Qualified PCR products were evaluated using an Agilent 2100 Bioanalyzer (Agilent, United States) and Illumina library quantitative kits (Kapa Biosciences, Woburn, MA, United States), which were further pooled together and sequenced on an Illumina NovaSeq 6000 (PE250).

Sequencing primer were removed from de-multiplexed raw sequences using cutadapt (v1.9). Paired-end reads were merged using FLASH (v1.2.8). The low-quality reads (quality scores < 20), short reads (<100 bp), and reads containing more than 5% “N” records were trimmed by using the sliding-window algorithm method in fqtrim (v0.94). Quality filtering was performed to obtain high-quality clean tags according to fqtrim. Chimeric sequences were filtered using Vsearch software (v2.3.4). DADA2 was applied for denoising and generating amplicon sequence variants (ASVs). The sequence alignment of species annotation was performed by QIIME2 plugin feature-classifier, and the alignment database was SILVA and NT-16S. Alpha and beta diversities were calculated using QIIME2. Relative abundance was used in bacteria taxonomy. The wilcox test was used to identify the differentially abundant genus, and significances were declared at P < 0.05. LDA effect size (LEfSe, LDA≥3.0, P value < 0.05) was performed using nsegata-lefse. Other diagrams were implemented using the R package (v3.4.4).

### 2.5 ELISA assay

The concentrations of IL-1β, IL-6, IL-10, and TGF-β in ileal and brain tissues were quantified using ELISA kits according to the manufacturer’s instructions.

### 2.6 Transcriptomic analysis

RNA was extracted from brain tissues of LPS-treated mice (n = 6 biological replicates per group), and transcriptome sequencing was performed by Shanghai Biotree Biotechnology Co., Ltd. (http://www.biotree.com.cn/). Following mRNA enrichment, RNA concentration was measured by Nanodrop (OD260/280 and OD260/230 ratios), RNA integrity was assessed using the Agilent 5400, and double-stranded cDNA fragments of 250–300 bp were size-selected with AMPure XP beads. After PCR amplification and purification, strand-specific libraries were constructed and sequenced on an Illumina Novaseq platform with PE150 strategy, generating 150 bp paired-end reads. Raw data were filtered to obtain clean data for subsequent bioinformatics analysis, and data quality was evaluated by FastQC (v0.23.4). Reads were mapped to gene exons for genome annotation using Hisat2 (v2.1), and genes with fewer than 15 reads per sample were excluded from further analysis. Differentially expressed transcripts (DETs) were identified using the DESeq2 package (v1.38.3) in R software. Functional enrichment analysis was performed with the clusterProfiler package (v4.6.2). Genes and gene sets with false discovery rate (FDR)-adjusted p-values < 0.05 were considered statistically significant, determined by multivariate analysis of variance (MANOVA). FDR correction was conducted using the Benjamini–Hochberg method after edgeR and MANOVA tests.

### 2.7 Western blotting

Western blot analysis was performed according to established protocols (Scandura et al., 2022). Tissues stored at −80 °C were ground in liquid nitrogen and homogenized in RIPA lysis buffer supplemented with protease inhibitors. The homogenates were centrifuged at 12,000×g for 15 min at 4 °C, and the supernatants were collected. Total protein concentration was determined using a BCA protein assay kit (Thermo Fisher Scientific) following the manufacturer’s instructions. Equal amounts of protein were separated by 12% sodium dodecyl sulfate–polyacrylamide gel electrophoresis (SDS-PAGE) (Green et al., 2019) and transferred onto polyvinylidene fluoride (PVDF) membranes. Membranes were blocked with 5% skim milk in TBST for 1 h at room temperature, followed by overnight incubation at 4 °C with primary antibodies against MBP (1:2000, Abcam, United Kingdom), Iba-1 (1:500, Abcam, United Kingdom), occludin (1:500, Abcam, United Kingdom), and C5aR1 (1:500, Abcam, United Kingdom). After three washes with TBST, membranes were incubated with horseradish peroxidase (HRP)-conjugated goat anti-mouse IgG secondary antibody (1:10000, Proteintech, China) for 1 h at room temperature. Protein bands were visualized using enhanced chemiluminescence (ECL) detection and quantified with ImageJ software (v2.3.0, NIH, United States). Expression levels of target proteins were normalized to GAPDH as an internal loading control.

### 2.8 Statistical analyses

All data are presented as mean ± standard deviation (SD). Statistical analyses were performed using GraphPad Prism (version 9.5.1, United States). The *t*-test or one-way analysis of variance (ANOVA) was used for comparison between groups. P < 0.05 was considered statistically significant.

## 3 Results

### 3.1 Glycine alleviates the WMI and inhibits microglial activation in septic mice

H&E staining revealed that brains from the sham group exhibited well-preserved white matter architecture, characterized by tightly packed, regularly arranged myelinated fibers and intact oligodendroglial nuclei. In contrast, the sepsis group displayed severe white matter pathology, including disorganized fiber tracts, axonal swelling, vacuolar degeneration, oligodendroglial pyknosis, and loss of structural integrity. Glycine treatment significantly attenuated these degenerative changes in septic mice, preserving white matter organization and markedly reducing edema in axons and surrounding tissue (Figure 1D).

IHC results revealed that in a mouse model of sepsis, the expression level of MBP in the cerebral white matter region was significantly decreased, with its positive staining intensity notably weakened, which suggests myelin sheath loss or damage. After glycine treatment, the expression level of MBP was restored (p < 0.05), indicating that glycine may have a certain repair effect on the myelin sheath (Figures 1E,G,I,K).

The IHC analysis of Iba-1 showed significant variations among the groups. The paraventricular white matter in the sepsis group had a considerably higher rate of Iba-1-positive areas than the placebo group. The glycine treatment significantly decreased microglial activations, as indicated by a marked reduction in the Iba-1-positive area rate (p < 0.05). The findings indicate that glycine may have neuroprotective effects in septic mice by modulating microglial activation in white matter regions (Figures 1F,H,J,L).

Compared to the sham group, the levels of IL-6, IL-1β, IL-10 and TGF-β in the sepsis group and the placebo group were also significantly elevated (p < 0.01). No significant difference was observed between the sepsis group and the placebo group (p > 0.05). After glycine treatment, the levels of IL-6 and IL-1β in the brain tissue were significantly reduced compared to the placebo group (p < 0.01). Compared to the placebo group, TGF-β levels were also significantly reduced (p < 0.05), while IL-10 levels decreased but remained higher than those in the sham group (p < 0.05). These findings suggest that glycine regulates inflammatory factors in brain tissue. Glycine treatment enables brain tissue to maintain a certain level of anti-inflammatory compensatory capacity, thereby preserving inflammatory homeostasis (Figures 1M–P).

### 3.2 The role of glycine in the gut microbiota of septic mice

**FIGURE 2:**
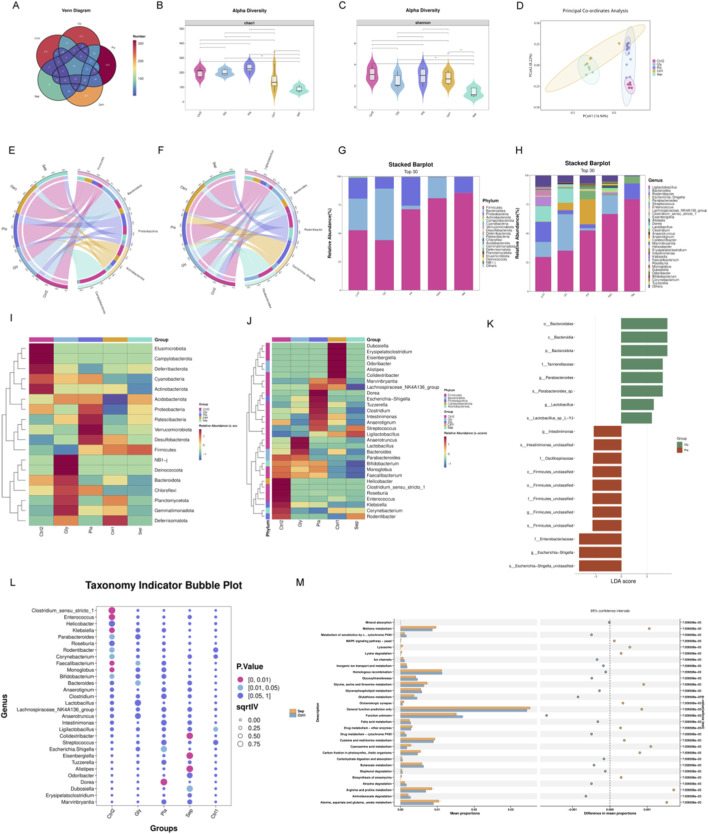
Glycine modulates gut microbiota in septic mice. **(A)** A Venn diagram showing the number of shared and unique ASVs in fecal samples from each mouse group. **(B,C)** Alpha diversity: Violin plots reveal Chao1 **(B)** and Shannon **(C)** index disparities between groups (**P < 0.01). **(D)** The PCoA analysis demonstrates beta diversity. **(E,F)** A circos plot showing the proportional distribution of the top five dominant species at the phylum **(E)** and genus **(F)** level. **(G,H)** A stacked bar chart representing species classification at the phylum **(G)** and genus **(H)** level. **(I,J)** Heatmap of the top 30 most abundant genera, organized by relative abundance at the phylum **(I)** and genus **(J)** level. **(K)** LEfSe genus level comparison of the Gly and Pla groups. **(L)** Indicator analysis of the top 30 most common genera across groups. **(M)** STAMP analysis for functional prediction reveals differences between the Sepsis and Sham groups. (Sep, Sepsis group; Gly, Glycine group; Pla, Placebo group; Ctrl1, sham group on P8; Ctrl2, sham group on P15; LEfSe, Linear Discriminant Analysis Effect Size); n = 5 per group).

We administered an orally gavaged glycine solution to septic mice for 7 days consecutively to investigate its effects on gut microbiota, followed by 16S rDNA sequencing of fecal samples (Figure 2). Figure 2A illustrates the Venn diagram depicting ASV distribution. The abundance and diversity indices of the gut microbiota significantly decreased in the sepsis group. To comprehensively assess the α-diversity of the gut microbiota in mice, we evaluated both the Chao1 and Shannon indices. Notably, following glycine intervention, septic mice exhibited improved microbial abundance and diversity compared to their pre-intervention state (Figures 2B,C). PCoA analysis of β-diversity revealed significant differences in the structure and community composition of gut microbiota across the groups (Figure 2D). Figures 2E–H depict the distribution of the five predominant microbial taxa at both the phylum and genus levels. Stacked bar charts at the phylum and genus levels illustrate notable differences in microbial composition across the groups (Figures 2G,H). Furthermore, these data are represented as heatmaps to facilitate a more detailed analysis (Figures 2I,J). At the phylum level, *Bacteroidota* predominated in the sham1 group; however, *Bacteroidota* decreased while *Proteobacteria* increased in both the sepsis and placebo groups. The glycine treatment group of the sham2 group demonstrated a sustained develop in *Bacteroidota* and a decrease in *Proteobacteria* compared to the placebo group. At the genus level, *Rodentibacter* and *Clostridium* exhibited an increase in the sepsis group relative to the sham1 group; conversely, *Bacteroides*, *Parabacteroides*, *Lactobacillus*, and *Eisenbergiella* showed a growing pattern in the glycine group compared to both the sham2 and placebo groups, whereas *Escherichia-Shigella* and *Rodentibacter* displayed a decline. These modifications signify a notable dysbiosis of intestinal microbiota in septic mice, with glycine able to restore beneficial microbiota and suppress harmful microbiota. LEfSe analysis revealed species exhibiting significant abundance differences between glycine-treated and untreated groups. After glycine treatment, in comparison to the untreated group, the abundances of *Bacteroidales*, *Parabacteroides,* and *Lactobacillus* were significantly increased. Conversely, in the untreated group, the levels of *Firmicutes*, *Enterobacteriaceae*, and *Escherichia - Shigella* were notably elevated (Figure 2K). The findings indicate that glycine can effectively improve gut microbiota dysbiosis in septic mice.

We conducted a detailed analysis of the 30 most abundant species to identify potential biomarkers. In the sepsis group, there was a significant increase in *Alistipes*, *Eisenbergiella*, and *Colidextribacter*. The administration of glycine resulted in a significant elevation of *Bacteroides* levels (Figure 2L). KEGG pathway analysis revealed that the MAPK signaling pathway, glycine/serine/threonine metabolism, and cysteine/methionine metabolism were significantly upregulated in the sepsis group compared to the sham group (Figure 2M).

### 3.3 Intestinal pathological changes in septic mice under glycine intervention

**FIGURE 3:**
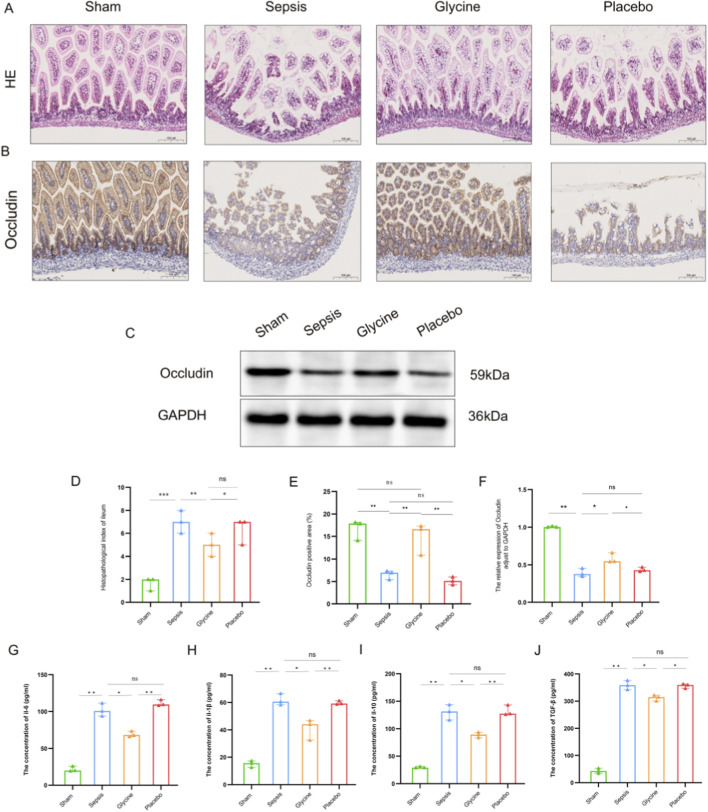
Glycine alleviates intestinal injury in septic mice. **(A)** Representative HE stained ileal tissues from each experimental group. **(B)** IHC staining of occludin in ileal tissues. **(C)** Western blot analysis of occludin protein expression in ileal tissues. **(D)** Histopathological injury scores of ileal tissues. **(E)** Quantification of occludin-positive area by IHC. **(F)** Densitometric analysis of occludin protein levels by western blot (normalized to GAPDH). **(G–J)** Concentrations of inflammatory cytokines IL-6 **(G)**, IL-1β **(H)**, IL-10 **(I)**, and TGF-β **(J)** in ileal tissues, measured by ELISA. Data are presented as mean ± SD. For HE staining and IHC staining: magnification, 200×; scale bar, 100 μm *P < 0.05; **P < 0.01; ***P < 0.001; ns for no significant difference. Data are representative of three independent experiments.

Preliminary observations showed that septic mice began to display clinical signs of distress, including lethargy and tremors, within 12 h after LPS administration. Hematochezia occurs within 24–48 h after LPS modeling. Histopathological analysis indicates that septic mice exhibit severe intestinal tissue damage, characterized by mucosal bleeding, infiltration of inflammatory cells, and loss of epithelial cells. Although glycine treatment significantly reduced pathological changes, mucosal loss remained evident in the LPS-induced ileum. The sepsis group exhibited extensive mucosal erosion within the inflammatory lesions, along with dense infiltration of inflammatory cells. Glycine significantly enhanced the histological injury score induced by LPS (p < 0.05) (Figures 3A,D).

In the ileal tissue of LPS-induced septic mice, the expression of occludin protein in the intestinal mucosa was markedly diminished compared to the sham group, indicating impairment of the intestinal mucosal barrier. The positive localization of occludin protein transferred from the cell membrane to the cytoplasm, indicating potential malfunctions of the occludin protein. The expression of the positive area in the glycine group markedly increased, suggesting that glycine treatment effectively restored the impaired intestinal mucosa (p < 0.05) (Figures 3B,C,E,F).

After LPS induction, the concentrations of IL-6, IL-1β, IL-10 and TGF-β in the ileal tissues of the sepsis group and the placebo group were significantly increased compared to the sham group (p < 0.01), with no difference observed between the sepsis group and the placebo group (p > 0.05). 7 days after glycine treatment, the levels of IL-6 and IL-1β in the ileal tissue were significantly reduced compared to the placebo group (p < 0.01), indicating a marked attenuation of the pro-inflammatory response. Comparing to the placebo group, the levels of TGF-β and IL-10 decreased (p < 0.05), with both cytokines showing a synchronous trend. These findings suggest that glycine attenuates both hyperactive pro-inflammatory signaling and exaggerated anti-inflammatory compensation in the ileum, facilitating a balanced resolution of inflammation (Figures 3G–J).

### 3.4 Transcriptomic analysis of WMI in septic mice

**FIGURE 4:**
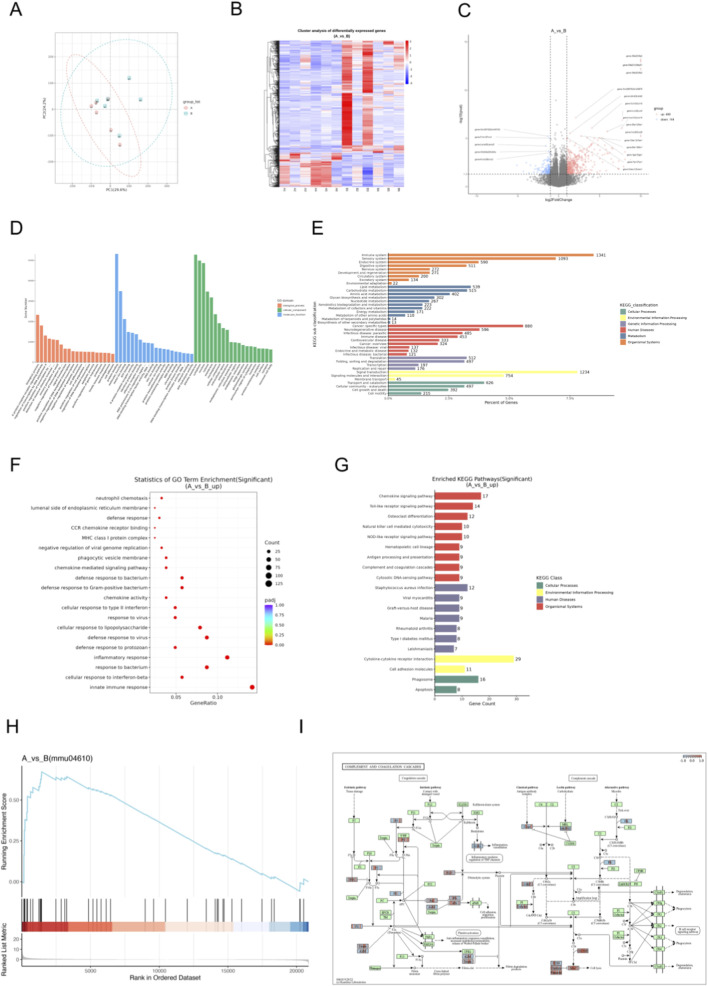
Transcriptomic screening of differential genes and signaling pathways in septic mice with WMI. **(A)** Principal component analysis, with red representing the sham group and the sepsis group. **(B)** Hierarchical clustering heatmap of differentially expressed genes (DEGs), where red and blue clusters indicate high and low expression levels in the septic and sham groups. **(C)** Volcano plot of DEGs, with red representing upregulated genes and blue representing downregulated genes. **(D)** GO annotation of DEGs. **(E)** KEGG annotation of DEGs. **(F)** Bubble plot showing GO enrichment of upregulated DEGs. **(G)** KEGG pathway visualization of upregulated DEGs. **(H)** GSEA plot for the complement and coagulation cascades pathway. **(I)** KEGG map of the complement and coagulation cascades pathway (n = 6 per group).

Transcriptome analysis of brain tissues from WMI mice and healthy controls identified differentially expressed genes (DEGs). Principal component analysis (PCA, Figure 4A) shows a separation between the two groups in the transcriptome space, suggesting that they have different global gene expression profiles. The hierarchical clustering heatmap (Figure 4B) displays differentially expressed genes (DEGs). The red and blue clusters represent high and low expression in the sepsis group and the placebo group respectively, demonstrating the substantial transcriptional changes associated with WMI. The volcano map (Figure 4C) shows all genes, with red dots indicating considerable upregulation, confirming the major DEGs contributing to individual segregation. The top ten differentially expressed genes in this study include Gm29879, Lcn2, Cxcl10, Zbp1, Lbhd2, C5aR1, Cxcl10, Lbhd2, Cxcl9, and Fpr1, all of which might play a role in the pathophysiology of sepsis-induced WMI.

GO and KEGG annotations allow for further investigation into the complex biological behaviors of genes. GO annotation describes the molecular functions (Molecular Function, MF), cellular components (Cellular Component, CC), and involved biological processes (Biological Process, BP) of genes (Figure 4D). KEGG annotation reveals that genes are primarily involved in the immune system, immune diseases, infectious diseases, signal transduction, signal molecules and interactions, as well as transport and catabolism (Figure 4E). GO Enrichment reflects that DEGs were enriched in biological processes (BP) such as innate immune response and defense response to bacterium (Figure 4F), reflecting robust immune activation in septic brain injury. Molecular function (MF) and cellular component (CC) enrichments supported dysregulated immune signaling (Figures 4F,G).

Figure 4H shows the KEGG gene set enrichment analysis (GSEA). Genes in the gene set are mainly concentrated near the peak of the enrichment score (ES) curve, indicating that these genes exhibit strong enrichment in gene regions highly relevant to the pathway. This suggests that the concentrated gene set has a positive correlation with the KEGG pathway, corresponding to an upregulated pathway.

According to the results of KEGG pathway enrichment, the Complement and coagulation cascades pathway was significantly enriched (Figure 4G, p < 0.05), a central mediator of septic inflammation and coagulopathy. Pathway mapping (Figure 4I) positioned C5aR1 at a key node, where its upregulation likely amplifies complement activation, aggravating inflammatory cascades and brain injury.

### 3.5 Verification of C5aR1 protein expression in the ileum and brain and tissues of septic mice

**FIGURE 5:**
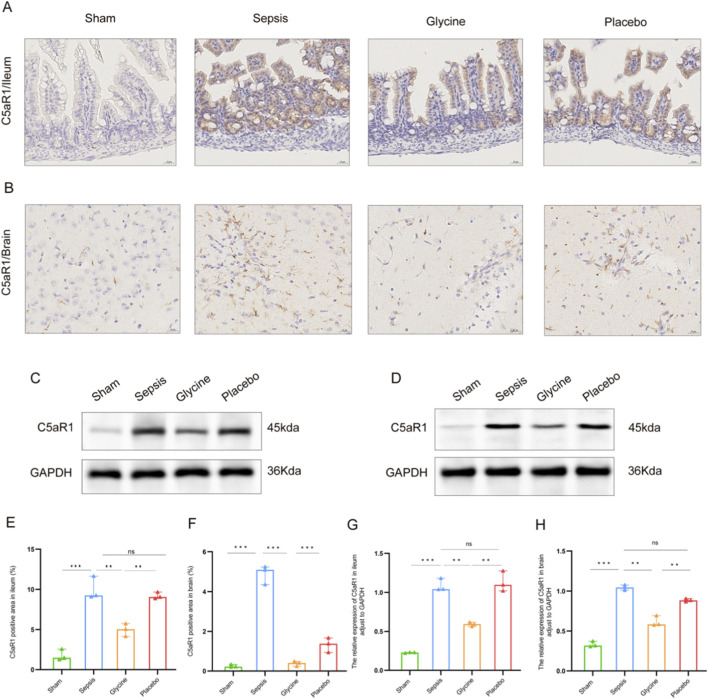
Significant expression of C5aR1 in the brain and ileum of septic mice. **(A,B)** Expression of C5aR1 in the ileum **(A)** and brain **(B)** tissues of mice by IHC. **(C,D)** Expression of C5aR1 protein in the ileum **(C)** and brain **(D)** tissues of mice shown by western blot. **(E,F)** Comparison of the positive area ratio of C5aR1 in the ileum **(E)** and brain **(F)** tissues by IHC. **(G,H)** Comparison of the densitometric analysis of C5aR1 protein in the ileum **(G)** and brain **(H)** tissues by western blot (normalized to GAPDH). For IHC staining: magnification, 400×; scale bar, 20 μm *P < 0.05; **P < 0.01; ***P < 0.001; ns for no significant difference. Data are representative of three independent experiments.

The expression of C5aR1 in intestinal mucosal epithelial cells of mice in the sepsis group was significantly upregulated (Figures 5A,C,E,G). The positive signals were mainly located in the cytoplasm and cell membrane of intestinal villus epithelial cells. The total positive area was significantly larger than that in the sham group. Glycine intervention significantly decreased C5aR1 immunoreactivity, resulting in a smaller positively stained region compared to the sepsis group (P < 0.05; Figures 5A,E). Western blot analysis revealed that the C5aR1 protein expression in ileum tissue increased significantly in the sepsis group and placebo group (P < 0.01), while in mice with glycine treatment, it decreased significantly (P < 0.05; Figures 5C,G).

Mice in the sepsis group had significantly higher levels of C5aR1 in their brain tissues, according to IHC analysis (Figures 5B,D,F,H). Both the cytoplasm and the cell membrane showed clear localization of the positive signals, which were mainly found in the neuroglial cells in the brain’s white matter region. After intervention with glycine, the positive expression of C5aR1 in the neuroglial cells of the brain white matter region was significantly weakened, the signal intensity in the cytoplasm and cell membrane decreased, and the positively stained area was significantly smaller than that in the sepsis group (P < 0.05; Figures 5B,F). These findings were corroborated by western blot analysis, which showed a significant increase in C5aR1 protein levels in the brain tissue of the sepsis group compared to the sham group (P < 0.01), and a significant reduction following glycine administration (P < 0.05; Figures 5D,H).

## 4 Discussion

Neonatal sepsis is reported to happen to 9.4% of all newborns in China (Cao et al., 2021). And WMI is the worse consequence for those who suffered from sepsis (Zorlular et al., 2025). In this study, we established a mouse model of sepsis and demonstrated that glycine exerts multiple protective effects: it attenuates white matter injury, suppresses microglial activation, restores gut microbial homeostasis, mitigates intestinal damage, enhances mucosal barrier integrity and function, and reduces pro-inflammatory cytokine levels in both the ileum and the brain. Additionally, transcriptome screening revealed that the coagulation cascade and complement pathways, along with the receptor C5aR1, might be significant mediators of sepsis-induced WMI. Notably, C5aR1 is markedly expressed in both the ileum and the brain, suggesting a potential role in mediating gut-brain crosstalk during sepsis. Although previous studies have investigated sepsis-associated organ damage (Zhang H. et al., 2024; Wang X. et al., 2023; Huo et al., 2023), this study represents the first systematic evaluation of the comprehensive protective effects of glycine.

The pathological changes of WMI are complicated, including the destruction of myelin sheath structure in white matter regions (Cao et al., 2023), the transformation of microglia from a quiescent state to an activated state, and the release of a large number of pro-inflammatory factors that exacerbate tissue damage (Zhou et al., 2024). As the main structural protein of myelin sheaths, MBP directly reflects the integrity of myelin sheaths. Abnormalities in its expression can indicate the status of myelin sheath injury or repair (Xie et al., 2024). As a specific marker of microglia, Iba-1 has its expression level closely associated with the activation degree of microglia, which can directly reflect the activity of inflammatory responses in the brain (Chen et al., 2024; Zhang L. et al., 2024). Our findings reveal that glycine may improve the destruction of MBP protein in septic mouse brain tissue, implying that glycine can reduce myelin reduction during sepsis-induced WMI. Furthermore, glycine inhibits microglial activation and decreases progressive inflammatory damage to the brain by down-regulating Iba-1 expression. Microglial activation may be either an inducer or a result of myelin damage: activated microglia release inflammatory factors that can directly damage oligodendrocytes and myelin sheaths, while fragments released from damaged myelin can further stimulate microglial activation, creating a vicious cycle (Shen et al., 2022; Fang et al., 2024). Our findings are consistent with previous studies on the anti-inflammatory and neuroprotective effects of glycine, and further reveal a novel mechanism by which glycine exerts dual protective actions in sepsis-associated white matter injury through modulation of the myelin-microglia interaction axis. Notably, recent studies have shown that TIGAR in macrophages drives inflammation by directly binding to TAK1 and promoting its ubiquitination and autophosphorylation; disrupting the TIGAR-TAK1 interaction significantly alleviates sepsis (Wang et al., 2024a). Although our study did not directly assess TAK1 activity, the inhibitory effects of glycine on pro-inflammatory cytokines and microglial activation suggest that it may indirectly modulate this pathway, warranting further investigation.

The gut microbiota and the metabolites have important biological implications for Immune regulation, inflammation, and neurological disorders (Wang et al., 2023b; Wang X. et al., 2024; Ren et al., 2025). The gut microbiota can dissociate bile acids, releasing taurine or glycine molecules (Milani et al., 2025). Glycine is an inhibitory neurotransmitter in the central nervous system that is involved in anti-inflammatory processes, immunological responses, and antioxidant responses (Meng et al., 2022; Xu et al., 2022). According to our investigation, glycine can regulate the gut microbiota of septic mice, and it not only preserves a healthy intestinal microecological homeostasis but also enhances the population of beneficial bacteria while suppressing the prevalence of harmful germs. Previous research has shown that glycine can improve the intestinal immunological barrier of piglets, alter the composition of intestinal bacteria, and improve mucoprotein content in the jejunum and ileum (Ji et al., 2022). It possesses the potential to maintain the mucosal barrier’s functionality, mitigate intestinal inflammation and allergies, and induce *Bacteroides acidifacens* to generate extracellular vehicles (EVs). Ultimately, alleviate the symptoms of irritable bowel syndrome (Zheng et al., 2023). While our results are consistent with prior research, the exact mechanism by which glycine regulates the gut microbiota in sepsis patients remains unclear and requires further investigation.

According to our findings, glycine can help septic mice recover from intestinal injury. In the glycine therapeutic group for sepsis, IHC analysis revealed significant repair of intestinal barrier integrity. Corresponding to this repair process, the expression level of the tight junction protein occludin was significantly increased; however, the cellular localization shifted from the functional cell-cell junction to a marked mislocalization and diffusion within the cytoplasm (Voulgaris et al., 2024; Fan et al., 2022; Feng et al., 2025). This alteration may have compromised its capacity to sustain epithelial barrier function. Functional impairment may arise from abnormal phosphorylation of occludin and subsequent cytoskeletal disorders, both induced by inflammatory factors and oxidative stress (Goncalves et al., 2022; Lu et al., 2022; Maridaki et al., 2025). Further analysis of occludin localization demonstrates that glycine preserves barrier integrity by inhibiting the abnormal translocation of the tight junction protein occludin from the cell membrane to the cytoplasm, mitigating LPS-induced intestinal injury. There is currently no evidence in the published literature indicating that glycine influences the subcellular localization of occludin. Our study reveals a novel mechanism by which glycine maintains intestinal barrier integrity.

Our study shows that sepsis induces inflammation in the ileum, along with a compensatory anti-inflammatory response. Glycine treatment significantly reduced pro-inflammatory cytokines (IL-6 and IL-1β) and also decreased anti-inflammatory cytokines (TGF-βand IL-10), suggesting that it restores intestinal immune homeostasis by modulating both pro- and anti-inflammatory arms of the immune response. These findings are consistent with prior studies showing glycine strengthens the gut barrier, reduces inflammation, and suppresses immune cell activation in the colon (Chen et al., 2021; Lee et al., 2023). In the brain, sepsis disrupts the blood-brain barrier, allowing peripheral inflammation to trigger microglial activation and neurodegeneration (Chmielarz et al., 2024). Glycine markedly decreased brain levels of IL-6, IL-1β, and TGF-β, while IL-10 remained relatively elevated compared to sham controls, indicating a rebalanced neuroinflammatory state. This aligns with earlier evidence that glycine protects against LPS-induced neuronal damage in young mice (Zhang et al., 2021). Our results demonstrate that glycine exerts dual protective effects in sepsis by simultaneously restoring intestinal immune homeostasis and rebalancing neuroinflammatory responses, highlighting its potential as a therapeutic agent targeting the gut-brain axis.

The complement system, a central component of innate immunity, is essential for maintaining homeostasis and defending against pathogens; however, its activation can also trigger excessive inflammatory responses (West and Kemper, 2023; Gavriilaki et al., 2022; Titiz et al., 2024). The interaction between C5a and its receptor, C5aR1, plays a key role in driving the inflammatory process (Zhu et al., 2025). After the complement system is activated, it generates C5a and binds to C5aR1, conjugates with the downstream heterotrimer Gi protein, activates downstream signaling pathways, recruits immune cells, and promotes the release of inflammatory mediators (Ye et al., 2024). Consistent with its aberrant expression in various infectious diseases, C5aR1 is markedly upregulated at both the mRNA and protein levels in organs such as the lungs, liver, kidneys, and heart during the early stages of sepsis, which is consistent with the abnormal expression of C5aR1 in various infectious diseases (Lu et al., 2025; Wang et al., 2023c). In experimental models of meningococcal infection, C5aR1-deficient mice display increased resistance to sepsis, whereas C5aR1 activation exacerbates disease progression (Muenstermann et al., 2019). In murine models of systemic fungal infection, the C5a-C5aR1 axis is essential for controlling *Candida* species, as evidenced by the significantly increased mortality and markedly elevated fungal burden observed in C5aR1 knockout mice resulting from infection (Desai et al., 2023). In our study, transcriptomic analysis revealed significant upregulation of C5aR1 in the brains of septic mice. We confirmed elevated C5aR1 expression in both brain and small intestine, which was markedly reduced by glycine treatment. These findings, building on prior evidence that C5aR1 drives sepsis-related organ injury, suggest that glycine protects against white matter injury, neuroinflammation, and intestinal damage by inhibiting the C5aR1-mediated complement pathway. In summary, glycine appears to exert multifaceted protective effects in sepsis by specifically targeting the C5aR1-mediated complement activation pathway in both the central nervous system and the gut. This finding not only deepens our understanding of glycine’s anti-inflammatory mechanisms but also offers a promising therapeutic strategy for sepsis-associated multi-organ injury.

There are still some limitations in this study. Firstly, this study mainly focuses on the WMI mechanism of bacterial sepsis, so the LPS-induced sepsis mouse model is adopted. This model accurately simulated the main pathological features of sepsis, but it did not fully capture the complexity of the clinical sepsis scenario or the diversity of pathogens, including bacterial, viral, and fungal sepsis, as well as the disease states of patients. We are currently unable to undertake parallel verification with various sepsis models due to experimental restrictions. Future study integrating diverse model types will provide a better understanding of glycine’s functional characteristics. Secondly, there is currently only relevant evidence supporting the link between glycine-induced gut microbiota modulation and intestinal barrier restoration and neuroprotective benefits. The techniques utilized in experiments involving faecal microbiota transplantation (FMT) and sterile animal research are complex and highly challenging. This study’s findings provide important insights into the potential connection and establish a foundational basis for future research on mechanisms. Thirdly, the specific mechanism of the metabolites of the gut microbiota in regulating glycine-mediated inflammatory responses has not been fully elucidated. However, emerging evidence indicates that these metabolites are involved in the regulation of the gut-brain axis (Sun et al., 2024; Zhuang et al., 2024). Notably, the simultaneous improvement of intestinal and brain white matter inflammation provides strong indirect support for this hypothesis. Fourthly, the transcriptomic analysis revealed that C5aR1 is a key mediator of septicemia-related brain injury, and its expression can be regulated through glycine intervention. However, the precise molecular mechanisms that control the interaction between C5aR1 and the gut microbiota remain to be clarified. This is also the content of our next research. However, the coordinated expression patterns of C5aR1 in the intestinal and brain tissues provide compelling preliminary evidence supporting the functional relationship between them.

In conclusion, glycine performed protective effects in sepsis. Glycine mitigates sepsis-induced WMI by modulating dysfunctional intestinal microbes. Our findings provide novel insights into the modulation of the gut-brain axis in sepsis and highlight the therapeutic potential of glycine. Glycine presents a potential therapeutic strategy for the prevention and treatment of brain injury induced by sepsis. This work has established a substantial theoretical framework that will serve as a valuable guide for future research in the field.

## Data Availability

The data presented in the study are deposited in the Zenodo repository, doi: 10.5281/zenodo.17976126
