# Supplementary figures and images for: Glycine attenuates sepsis-induced white matter injury by modulating gut microbiota
Source: Front Mol Biosci. 2026 Jan 8;12:1733207. doi: 10.3389/fmolb.2025.1733207 (PMC12824023; doi:10.3389/fmolb.2025.1733207)

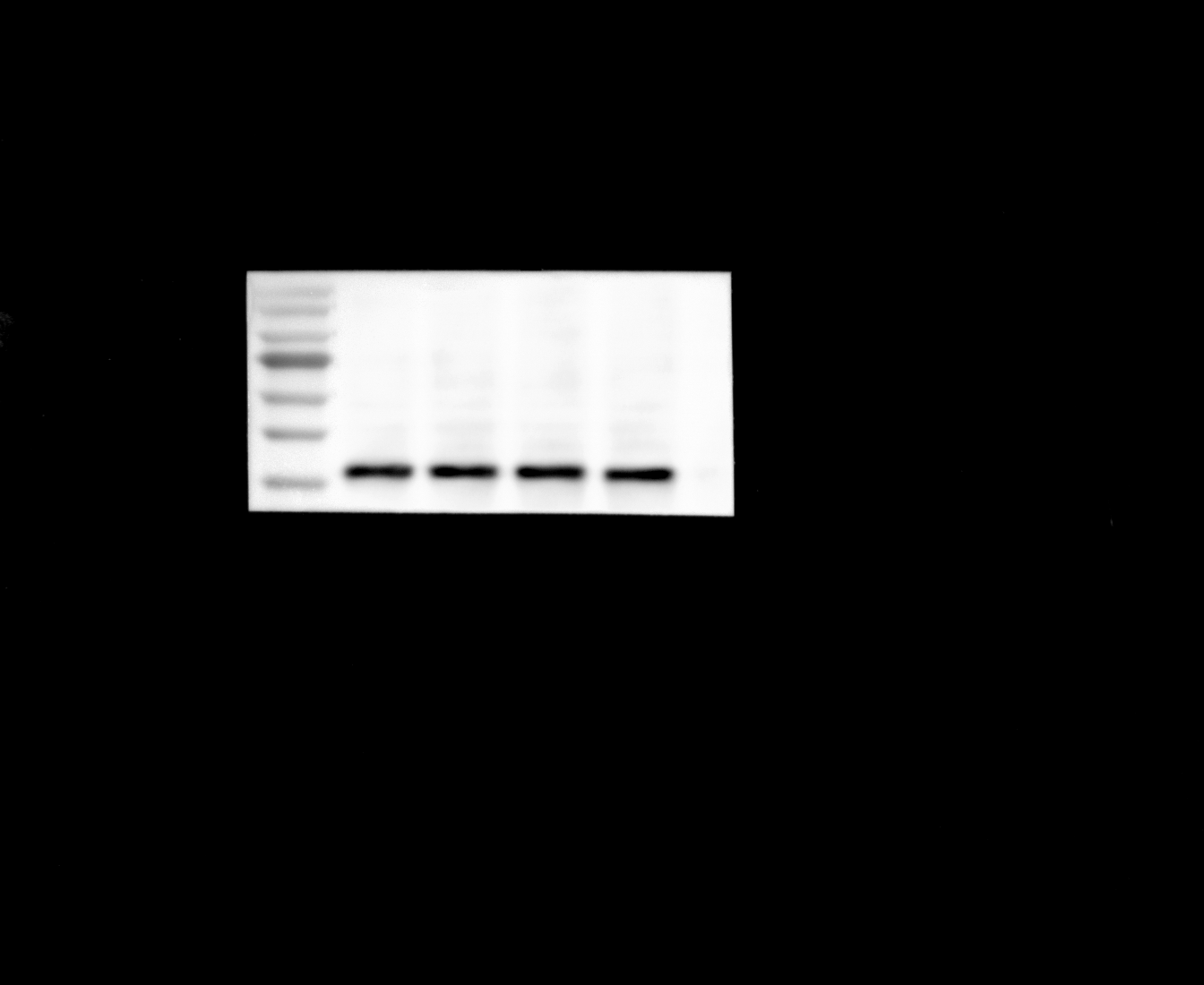

Supplement: Supplementary file 1 [file Supplementaryfile1.zip › WB/Figure1 G GAPDH.Tif]

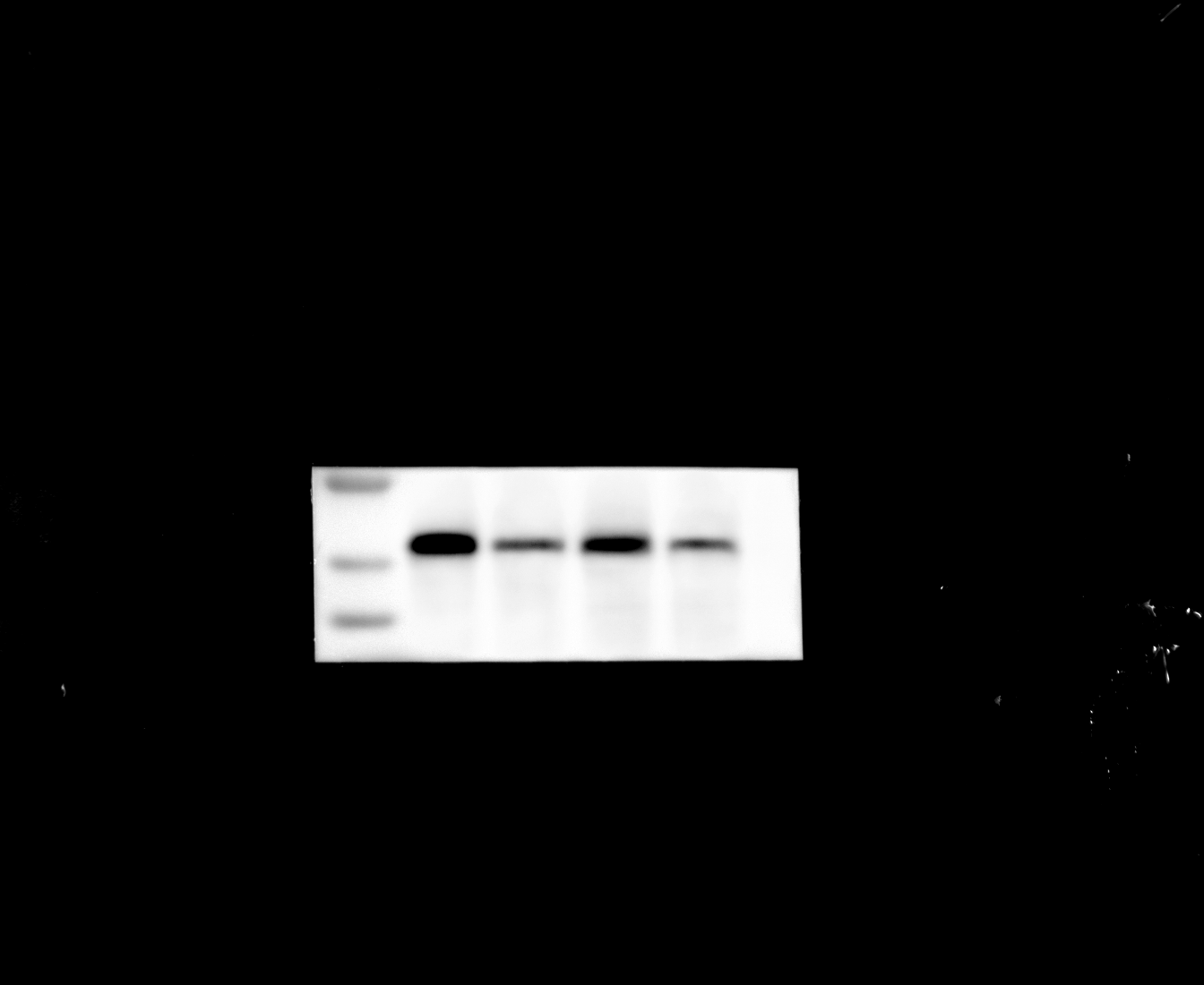

Supplement: Supplementary file 1 [file Supplementaryfile1.zip › WB/Figure1 G MBP.Tif]

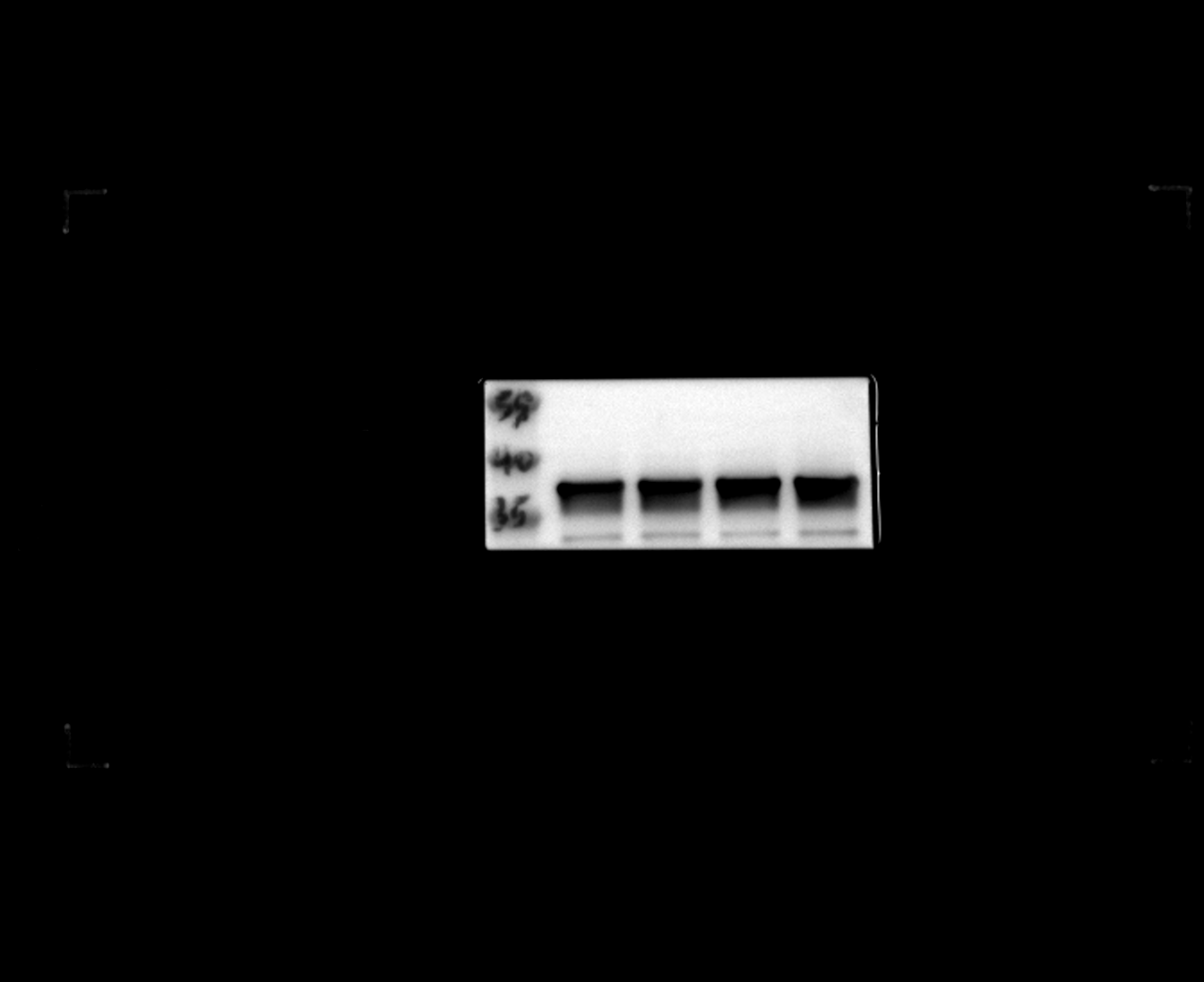

Supplement: Supplementary file 1 [file Supplementaryfile1.zip › WB/Figure1H-GAPDH.Tif]

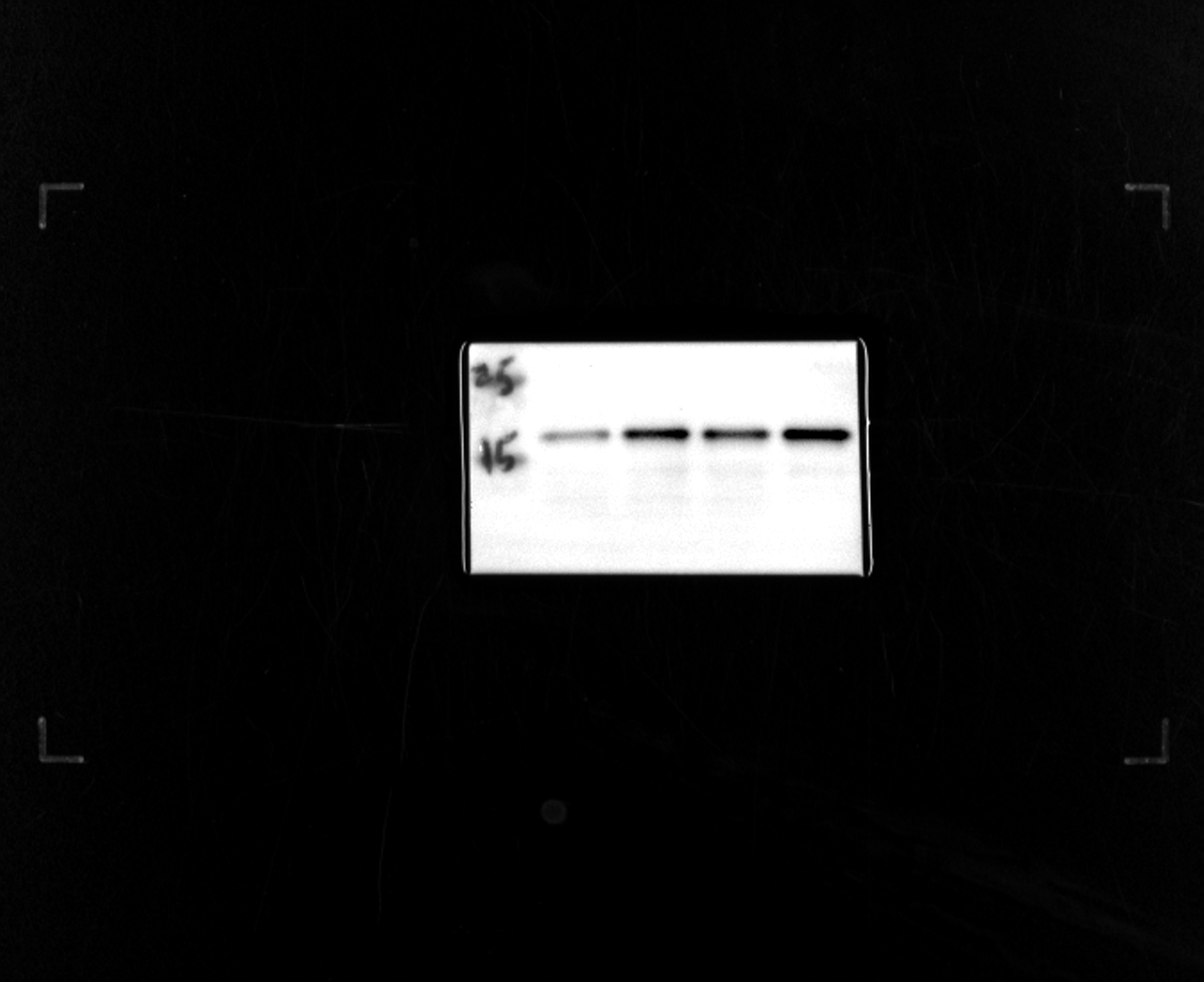

Supplement: Supplementary file 1 [file Supplementaryfile1.zip › WB/Figure1H-IBA1.Tif]

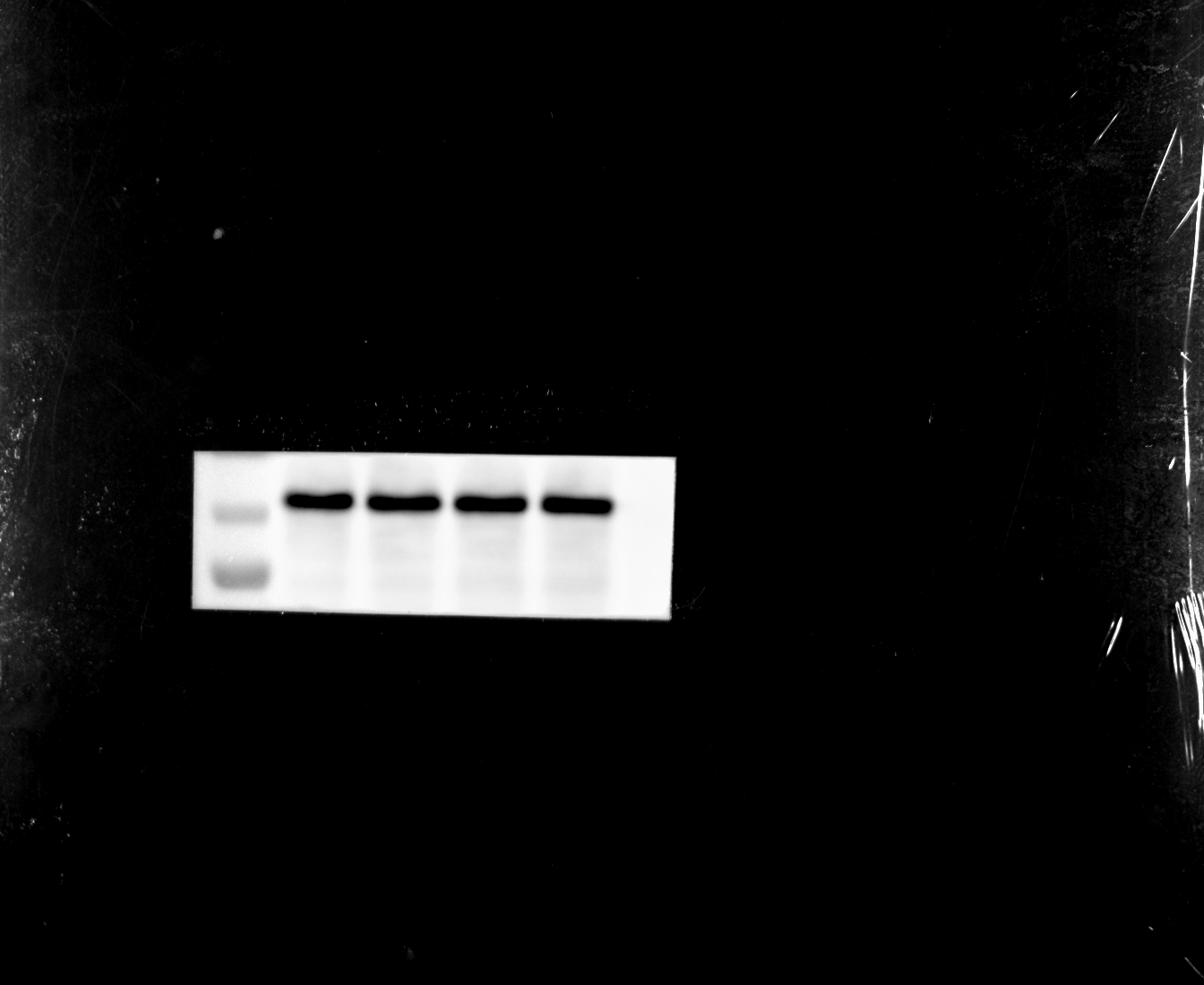

Supplement: Supplementary file 1 [file Supplementaryfile1.zip › WB/Figure3C-GAPDH.Tif]

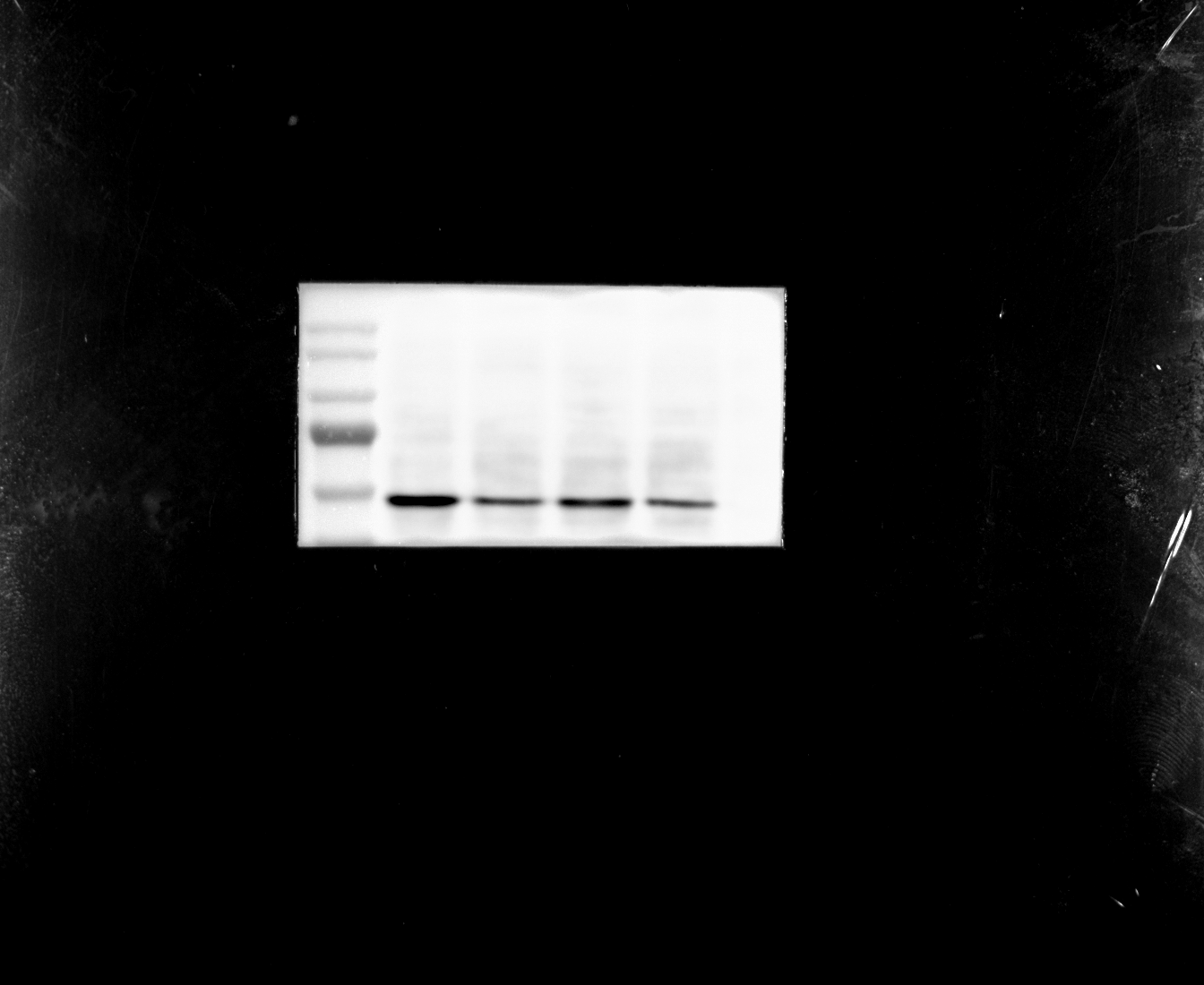

Supplement: Supplementary file 1 [file Supplementaryfile1.zip › WB/Figure3C-Occludin.Tif]

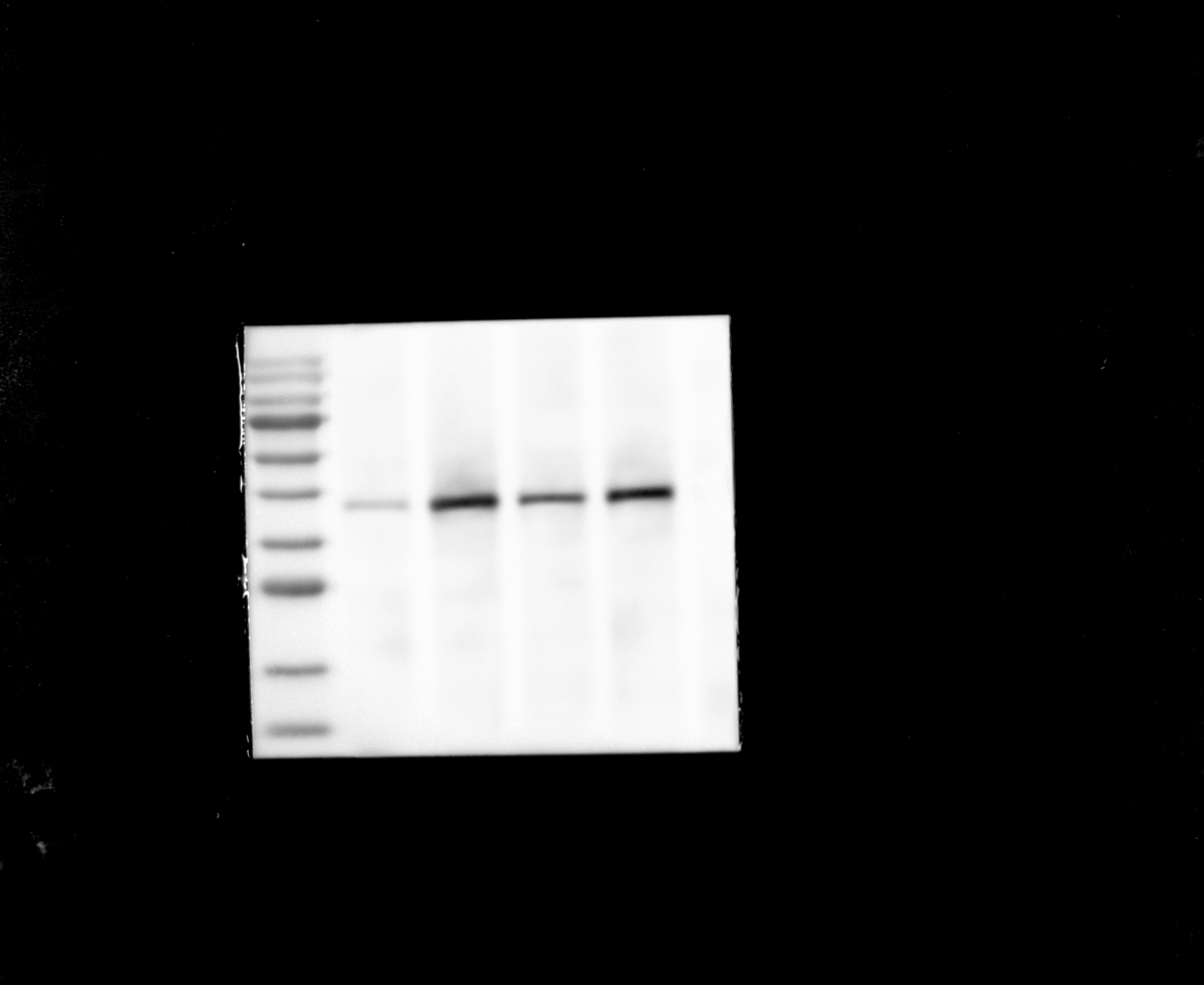

Supplement: Supplementary file 1 [file Supplementaryfile1.zip › WB/Figure5C-C5ar1.Tif]

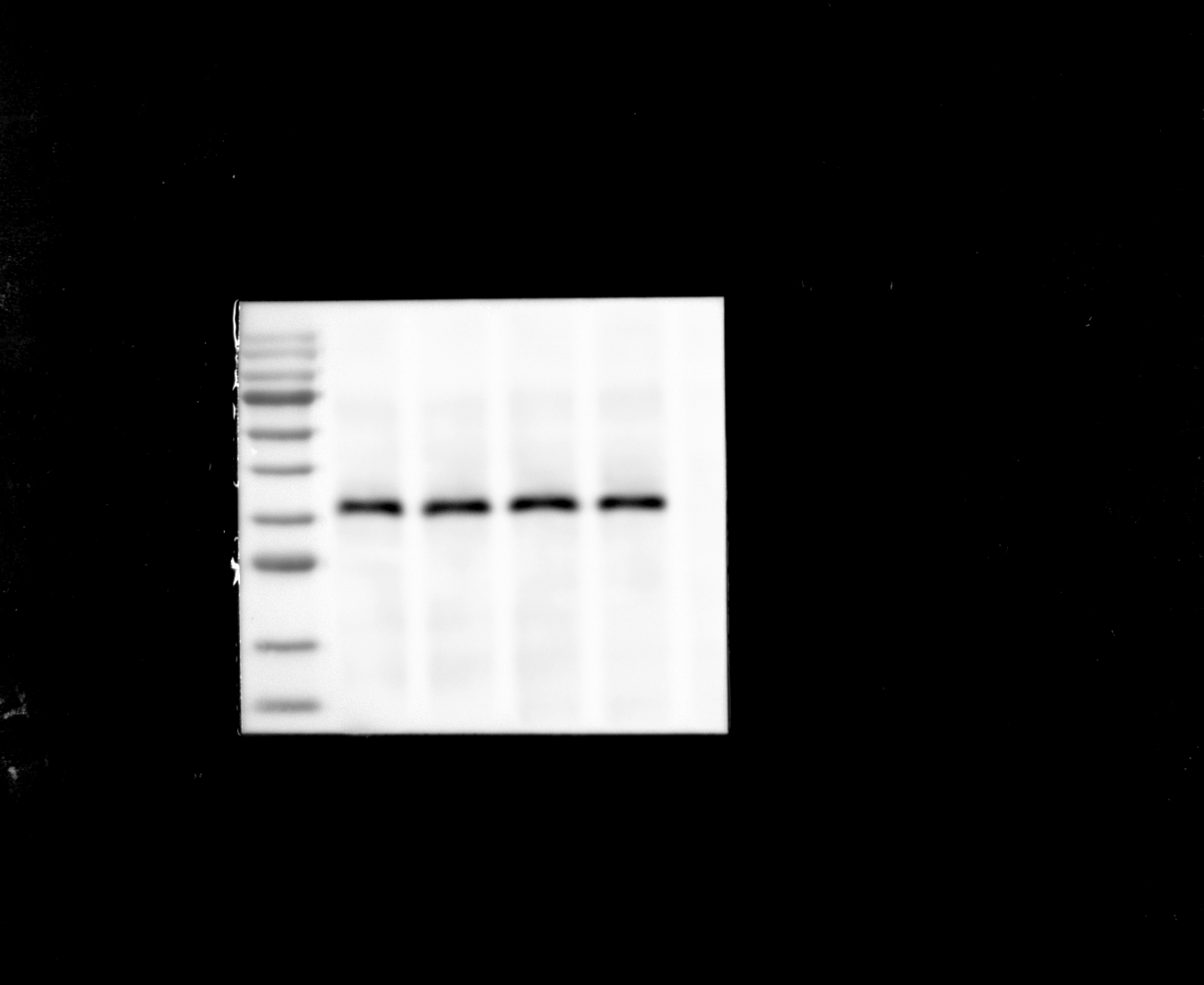

Supplement: Supplementary file 1 [file Supplementaryfile1.zip › WB/Figure5C-GAPDH.Tif]

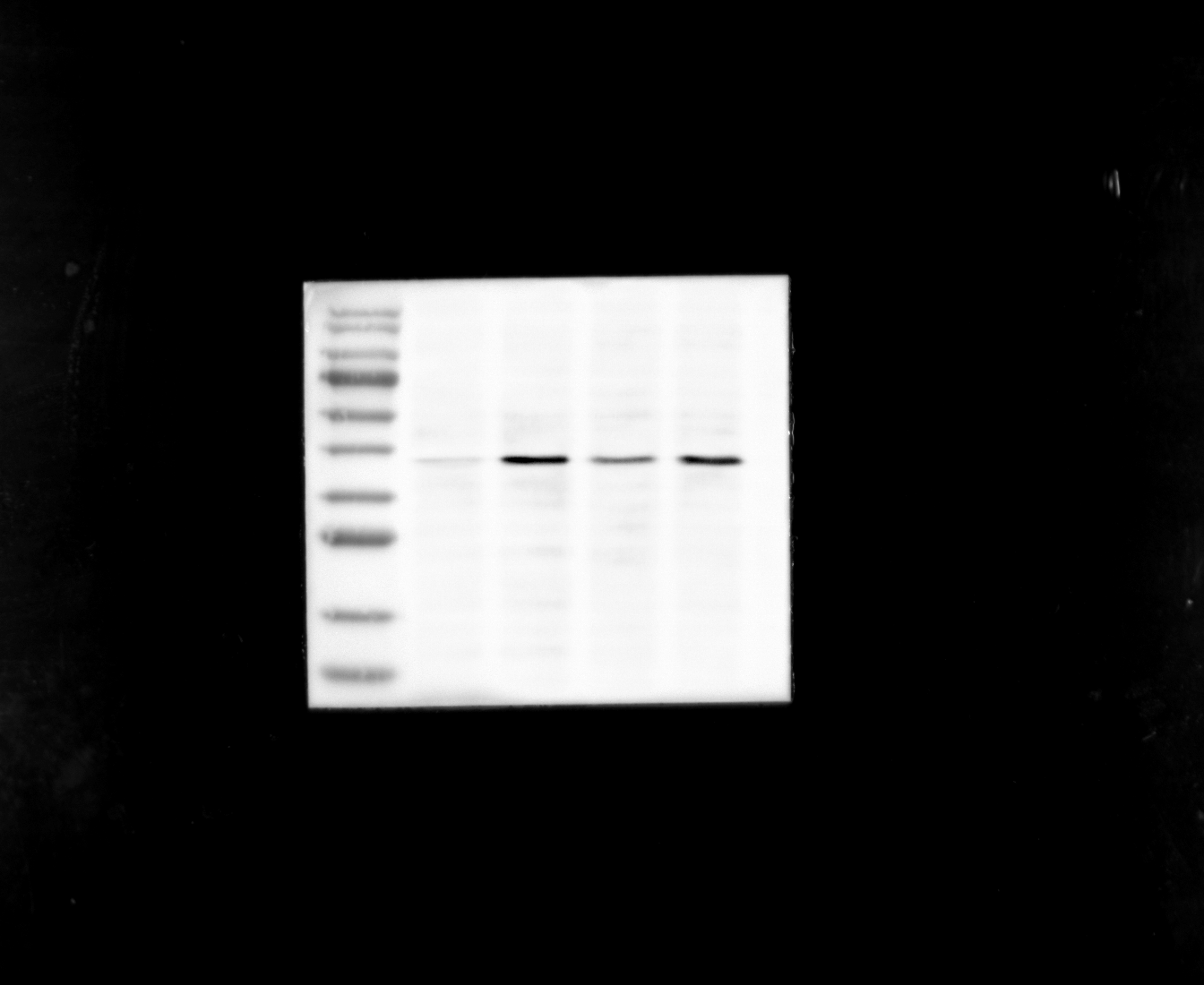

Supplement: Supplementary file 1 [file Supplementaryfile1.zip › WB/Figure5D-C5ar1.Tif]

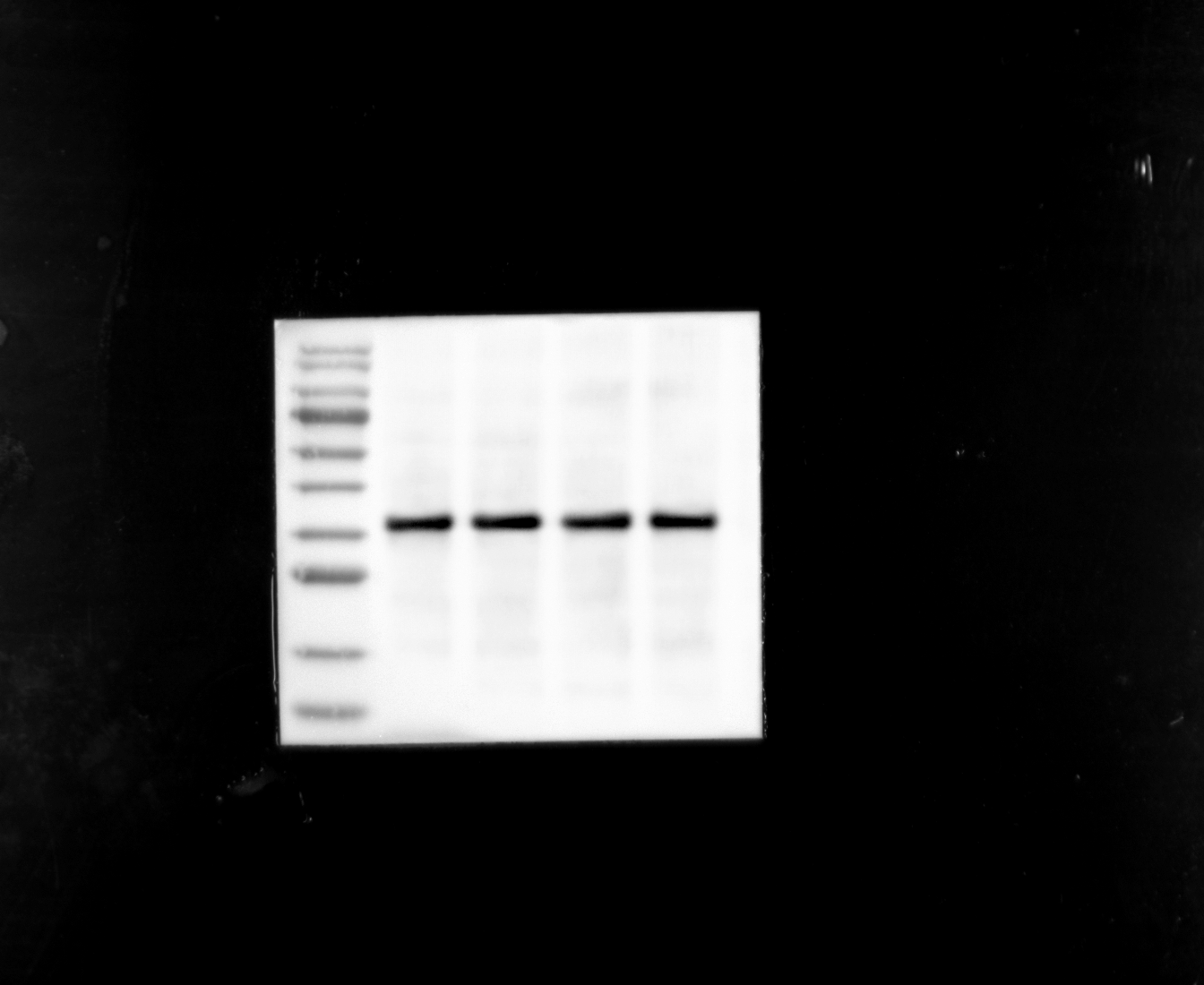

Supplement: Supplementary file 1 [file Supplementaryfile1.zip › WB/Figure5D-GAPDH.Tif]
